# WS_2_: A New Window Layer Material for Solar Cell Application

**DOI:** 10.1038/s41598-020-57596-5

**Published:** 2020-01-21

**Authors:** Md. Khan Sobayel Bin Rafiq, N. Amin, Hamad F. Alharbi, Monis Luqman, Afida Ayob, Yahya S. Alharthi, Nabeel H. Alharthi, Badariah Bais, Md. Akhtaruzzaman

**Affiliations:** 1 Solar Energy Research Institute, The National University of Malaysia, 43600 Bangi, Malaysia; 2 Department of Electrical, Electronic and Systems Engineering, Faculty of Engineering and Built Environment, The National University of Malaysia, 43600 Bangi, Selangor Malaysia; 3 Institute of Sustainable Energy, Universiti Tenaga Nasional (@The National Energy University), Jalan IKRAM-UNITEN, 43000 Kajang, Selangor Malaysia; 4 Mechanical Engineering Department, King Saud University, P.O. Box 800, Riyadh, 11421 Saudi Arabia; 5 Center of Excellence for Research in Engineering Materials, King Saud University, Riyadh, 11421 Saudi Arabia

**Keywords:** Materials science, Renewable energy

## Abstract

Radio frequency (RF) magnetron sputtering was used to deposit tungsten disulfide (WS_2_) thin films on top of soda lime glass substrates. The deposition power of RF magnetron sputtering varied at 50, 100, 150, 200, and 250 W to investigate the impact on film characteristics and determine the optimized conditions for suitable application in thin-film solar cells. Morphological, structural, and opto-electronic properties of as-grown films were investigated and analyzed for different deposition powers. All the WS_2_ films exhibited granular morphology and consisted of a rhombohedral phase with a strong preferential orientation toward the (101) crystal plane. Polycrystalline ultra-thin WS_2_ films with bandgap of 2.2 eV, carrier concentration of 1.01 × 10^19^ cm^−3^, and resistivity of 0.135 Ω-cm were successfully achieved at RF deposition power of 200 W. The optimized WS_2_ thin film was successfully incorporated as a window layer for the first time in CdTe/WS_2_ solar cell. Initial investigations revealed that the newly incorporated WS_2_ window layer in CdTe solar cell demonstrated photovoltaic conversion efficiency of 1.2% with V_oc_ of 379 mV, J_sc_ of 11.5 mA/cm^2^, and FF of 27.1%. This study paves the way for WS_2_ thin film as a potential window layer to be used in thin-film solar cells.

## Introduction

For many decades, transition metal dichalcogenides (TMDCs) are among the promising materials in a wide range of applications, but their superior performance in complex applications has attracted extensive research attention. TMDCs, particularly MoS_2_ and WS_2_, have recently raised particular concerns for the photovoltaic community^1,2^ due to their suitable bandgaps in the range of 1–2 eV and high absorption coefficient of over 10^5^ cm^−1^ ^3–7^. Although WS_2_ is more abundant in the Earth’s crust, cheaper, and less toxic compared to other TMDC materials, the development of WS_2_ in thin-film solar cells remains in its infancy compared with similar photovoltaic materials. Very recently, tungsten disulfide (WS_2_) has become the focus of thin-film solar cell materials due to its opto-electrical properties. Although the individual crystals of this material have been studied in optical devices, only a few studies have been carried out concerning the photovoltaic properties of a thin film. An important characteristic that is seldom addressed is its tunable bandgap. WS_2_ can exhibit a high direct bandgap (>2 eV) and a low indirect bandgap (<1.5 eV) depending on its fabrication technique^8^. It has a considerably large size of W atoms that can tailor its structural properties based on its application^8,9^.

A typical thin-film solar cell structure comprises a highly doped coating on a substrate where a similar but moderately doped absorber layer is deposited on top of it. Above the absorber layer, a heavily but opposite conductive material is doped that functions as an emitter or window layer^7^. The window layer in a heterojunction thin-film solar cell is primarily used to form a p-n junction with the absorber layer. This layer is desired to achieve a high bandgap, small thickness, and low series resistance for high optical throughput. Absorber layers must have high optical absorption coefficient with high mobility, good carrier lifetime, and enhanced crystallographic properties^10,11^. CdS is widely used as a window or buffer layer material in photovoltaic devices due to its suitable bandgap and enhancement properties in the interface chemistry between light absorber and window layer during fabrication. However, the toxicity of Cd^12^ and hazardous impact of CdCl_2_ treatment during fabrication^13^ of CdTe-based solar cells are the major disadvantages of CdS. Thus, researchers have sought to investigate other materials for window layer application. Several environmentally friendly, wide bandgap materials such as ZnS, ZnSe, and ZnO are currently being investigated to replace CdS^10,14,15^. Researchers have yet to find an ideal window layer material to replace toxic CdS.

TMDC thin films for photovoltaic application are grown by a variety of methods. At least two groups deposited WS_2_ and MoS_2_ by chemical vapor deposition (CVD). In both the cases, MoS_2_ films exhibited good photoconductivity, while WS_2_ did not. WS_2_ films have also been deposited from tungsten by sulfurization^16,17^ or sulfurizing tungsten oxide films^17,18^ or from tungsten targets by reactive magnetron sputtering in Ar/H_2_S atmosphere^19–21^ or by electrodeposition^22^; however, incorporating WS_2_ for photovoltaic applications has yet to be achieved. As-deposited films were found sulfur-deficient and exhibited poor crystallographic quality, a porous morphology, and a high defect density^23,24^. Seegar *et al*. opined that low sputtering pressures with increased particle bombardment (deposition power) of the growing film causes significant changes in morphology but increases defect density and incorporates impurities to films; these impurities need to be addressed when the films are used for solar cells^25,26^. As radiofrequency (RF) magnetron sputtering is a plasma- or ion-assisted deposition process, the slightest variation in deposition parameters may cause changes in structure and bonding of the deposited material^27,28^. Such changes may lead to both advantageous and detrimental effects for photovoltaic application. For this reason, deposition parameters play a wide role in the preferential growth process in sputtering. Thus, optimization of growth parameters in RF magnetron sputtering is crucial. To investigate the suitable application of WS_2_ in solar cells, we extensively studied the morphological, structural, and optoelectrical properties of WS_2_ deposited by RF magnetron sputtering under different deposition powers. Finally, a CdTe-WS_2_ complete thin-film solar cell was successfully fabricated using WS_2_ as a window layer for the first time. The study also aimed to eliminate pinholes in sputtered films to improve the stability and efficiency of the solar cell device.

## Methodology

### WS_2_ thin-film deposition

WS_2_ thin film was deposited by RF magnetron sputtering deposition, which is one of the prominent physical vapor deposition techniques under high vacuum condition. In this study, deposition was carried out on soda lime glass substrates of 7.5 cm × 2.5 cm × 0.2 cm. They were pre-cleaned by sequential cleaning, such as mechanical scrubbing, followed by acetone–methanol–deionized water in an ultrasonic bath and later dried with N_2_ gas flow. A 50 mm diameter WS_2_ (99.99%) sputtering target (supplied by Kurt. J. Lesker) was used as the source material. Initially sputtering chamber was purged twice to remove unwanted components from the chamber. The chamber’s base pressure was brought down to 10^−6^ torr by a turbomolecular pump. During deposition, the working pressure was maintained at 10^−2^ torr to instigate the plasma. The target-to-substrate distance (sputter down) and substrate holder rotation were fixed at 8 cm and 10 rpm, respectively. Deposition parameters used for this study are shown in Table 1. During the deposition process, the substrate temperature was fixed at 100 °C. All the samples were stored inside the sputtering chamber upon completion of deposition until the substrate temperature fell to room temperature to prevent as-grown films from being oxidized.

**Table 1:** Sputtering Parameters.

Parameter	Condition/Value
Target	WS_2_ (99.9% pure)
Substrate	Soda Lime Glass
Base Pressure	3.0 × 10^−6^ Torr
Working Pressure	2.1 × 10^−2^ Torr
Growth Temperature	100 °C
RF Power	50 W, 100 W, 150 W, 200 W & 250 W
Gas & Flow Rate	Argon (4.0 sccm)

### Full device fabrication

**Figure 1:**
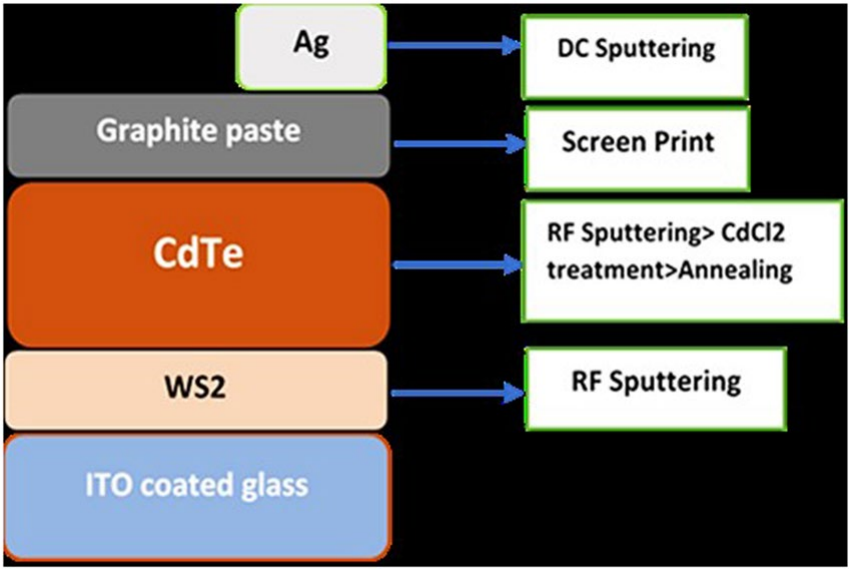
Device structure with fabrication process of CdTe/WS_2_ solar cells.

The optimized WS_2_ thin film was incorporated as a window layer in lieu of CdS in CdTe solar cell. For the initial study, the basic superstrate structure of the CdTe solar cell was chosen. Full device fabrication included the stack of five layers, namely, indium tin oxide (ITO) for the transparent conducting oxide, the n-type WS_2_ window layer, the p-type absorber CdTe, Cu-doped graphite paste for improved ohmic contact, and silver (Ag) as back contact. The complete cell was fabricated by sputtering as reported by previous researchers^29^. Figure 1 represents the structure and full cell device fabrication process.

### Film characterization

The structural and crystalline properties of the as-grown films were examined by a BRUKER aXS-D8 Advance CuKα diffractometer at room temperature. X-ray diffraction (XRD) patterns were recorded in the 2ϴ range of 10°–80° with a step size of 0.05° using Cu Kα radiation wavelength λ = 0.15408 nm. Atomic force microscopy (AFM) was used to examine the surface pattern and roughness of the film. Grain size, surface morphology, and cross-sectional views were observed by using a Carl Zeiss Merlin field-emission scanning electron microscope (FESEM) operated at 3 kV. Electronic properties such as carrier concentration, mobility, and resistivity were measured by a Hall Effect measurement system (HMS ECOPIA 3000) with a magnetic field of 0.57 T and probe current of 100 nA for all the samples. Characterization on optical properties was carried out to determine the absorbance, transmittance, and optical bandgap. These measurements were performed at room temperature by using a Perkin Elmer Lambda 950 UV-Vis-NIR spectrophotometer. The absorption coefficient and bandgaps were calculated from Tauc plots. Pinholes were investigated by keeping as-deposited samples over a light source, and the adhesiveness of films was tested by the “Scotch tape” method. To evaluate the performance of full cells, newly fabricated cells underwent light I-V testing. The light source in the I–V tester replicated the standard 1.5 AM G spectrum. Dark I–V data were also obtained using the same equipment.

## Results and Discussion

### Adhesiveness of film

Adhesiveness is a pre-requirement for any deposition on substrate. The Scotch tape method is an effective technique to check the adhesiveness of any film^30^. The Scotch tape test is usually conducted on samples immediately after the deposition process. Thus, it was performed after the natural cooling of substrates. Table 2 shows the outcome of the Scotch tape test with respect to different powers. Most of the films showed good adherence to the SLG substrates, except for the few films that were deposited at high deposition power. Some elemental WS_2_ was observed at deposition of 250 W. In principle, sputtering is the bombardment of particles/species with high energy. A threshold energy for the discharge of atomic species from the target material exists, below which the atom will not be “sputtered.” The yield will increase with the application of deposited power. However, if high input of power is superimposed with its default energy, it causes the bombarded particles’ with a very high rate and causes to bounce the particles from substrate. Therefore, at very high energies due to high deposition power, the yield of films decreases and reduces the adhesion of the film^31^.

**Table 2:** Adhesiveness Test Result.

Sputtering Target	Deposition Power (W)	Scotch tape Test
WS_2_	50	Pass
	100	Pass
	150	Pass
	200	Pass
	250	Not passed

### Pinhole elimination

**Figure 2:**
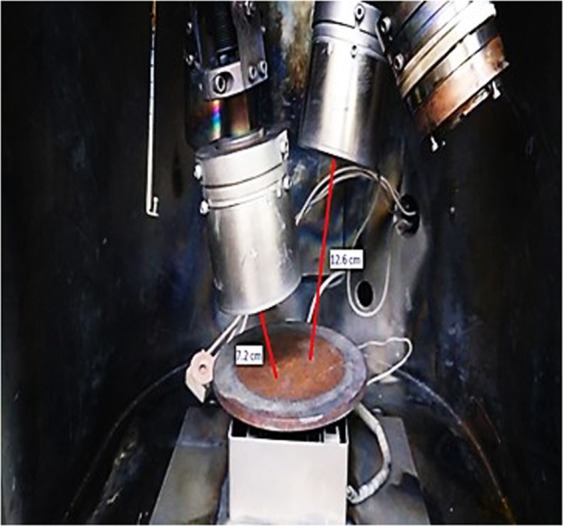
Physical view of the pinhole reduction technique.

Pinholes were observed by illuminating the metallic film on glass from behind, which sometimes referred to as pin windows. The metallic film will attenuate light when viewed from the front, except for a scattering of bright light pinpricks. The pinpricks of light are often almost circular in shape where there is an area of unmetallized film. These unmetallized areas are primarily caused by the metallization of dust or debris on the surface as the film passes through the deposition zone. However, sometimes after the debris is moved, an un-metallized area that corresponds to the debris’ shadow shape remains. Occasionally, the debris does not roll away but slides away, so the pinhole may also have a scratch track leading away from the unmetallized area. There are two types of pinholes; natural and artificial. Natural pinholes rarely occur and are difficult to eliminate as they come with the source material^32^. However, artificial pinholes can be eliminated easily by using different techniques. Figure 2 exhibits the pinhole reduction technique inside the sputtering chamber. In this particular experiment, pinholes were effectively removed by changing the substrate-to-target distance from 8 cm to 10.6 cm.

### Film morphology

**Figure 3:**
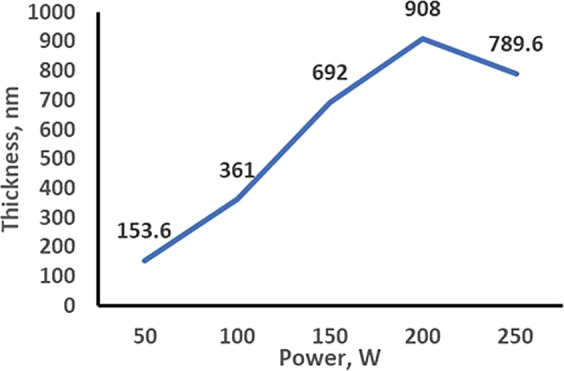
Thickness–power relationship for sputter grown WS_2_.

Figure 3 shows the thickness–power relationship of as-grown WS_2_ for different RF powers. The film thickness increased with the increase in deposition power. However, this phenomenon was only applicable up to 200 W in this study. Above this power, the film thickness was reduced because of the high energetic bombardment of sputtered atoms, which caused the atoms to bounce back from substrates.

**Figure 4:**
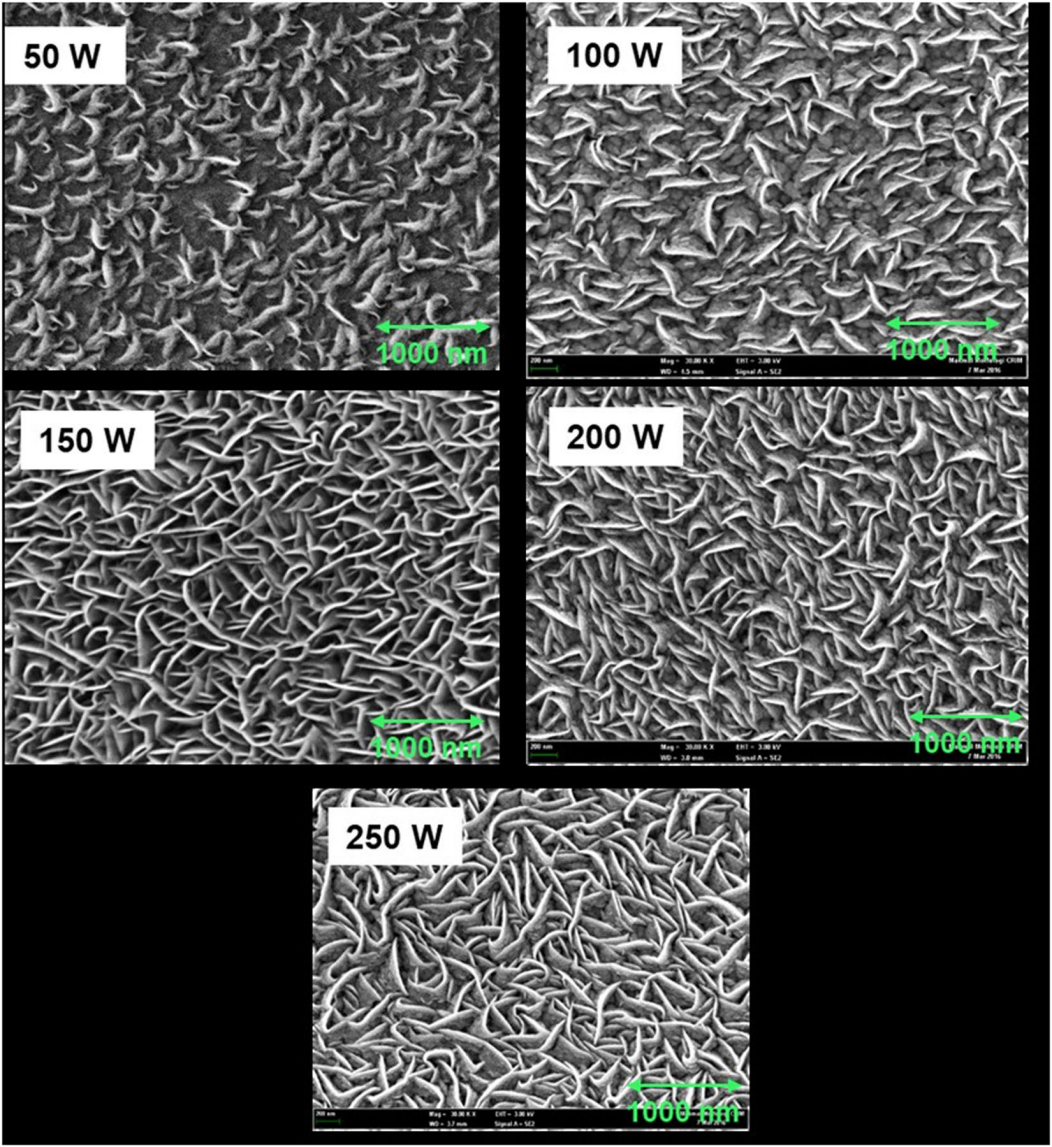
FESEM images of as-grown WS_2_ at 350 nm thickness for different deposition powers.

**Figure 5:**
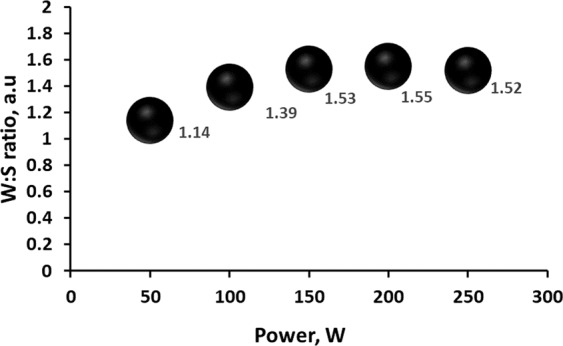
W:S ratio of as-grown WS_2_ for different deposition powers.

FESEM images of WS_2_ thin films presented in Fig. 4 reveal the surface morphologies of sputtered WS_2_ thin films at different deposition powers. In this case, the thickness of the film was retained at 350 nm. Our literature review indicated that films sputtered at low deposition power exhibit porous microstructures while films sputtered at high deposition power exhibit dense microstructures^33^. The number of particles received at the substrate increases with the increase in deposition power, thereby resulting in a dense microstructure. At low deposition power, the deposition rate decreases and the number of species arriving at the surface of the substrate is reduced, resulting in a porous microstructure. In this study, all the films showed a smooth morphology and revealed a dense microstructure for deposition powers of 100 W and above. However, no porous microstructure was observed in the case of WS_2_. Moreover, the as-grown WS_2_ thin films with different RF powers showed granular morphology. EDX analysis revealed that all the films exhibited a sulfur deficit (Fig. 5). The highest sulfur-to-tungsten ratio (1: 1.55) was found at 200 W deposition power. Grain size was calculated with ImageJ software^29^, and the results revealed that grain size increased with the deposition power. The highest grain size of 76.25 nm was found at 150 W deposition power, whereas the lowest of 4.86 nm was found at 50 W deposition power. The average grain size at a deposition power of 150 W and above was 26.74 nm. Thus, a deposition power of 150 W and above has insignificant effects on the grain size of WS_2_ thin film.

**Figure 6:**
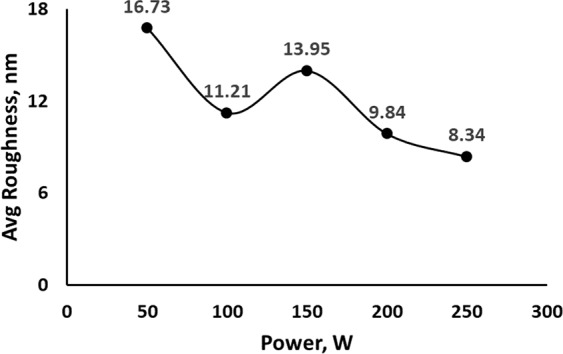
Average roughness of as-grown WS_2_ thin film.

**Figure 7:**
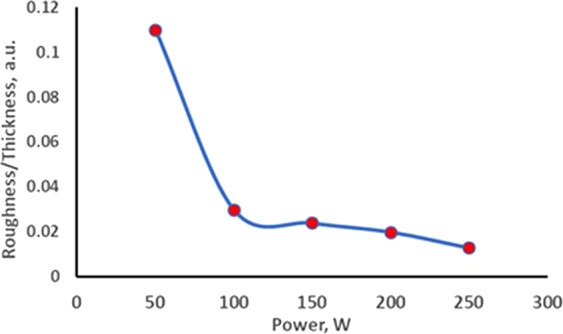
Roughness/thickness relationship for different RF powers.

The roughness of the deposited film was measured by atomic force microscopy (AFM). Figure 6 shows the average roughness of the deposited films. The film roughness decreased with the increase in power, but hardly any changes in roughness were observed after a certain level of input power. The ratio between roughness and thickness decreased with power due to the non-formation of droplets and increment of thickness. This ratio remained almost the same between 150 and 250 W. Figure 7 illustrates the relationship between roughness/thickness and power. The probability of absorbance increased when light waves were scattered. Low roughness on the film surface facilitates light waves to transfer easily without scattering. Thus, a high deposition power is necessary when considering WS_2_ as a window layer material.

### Structural properties

**Figure 8:**
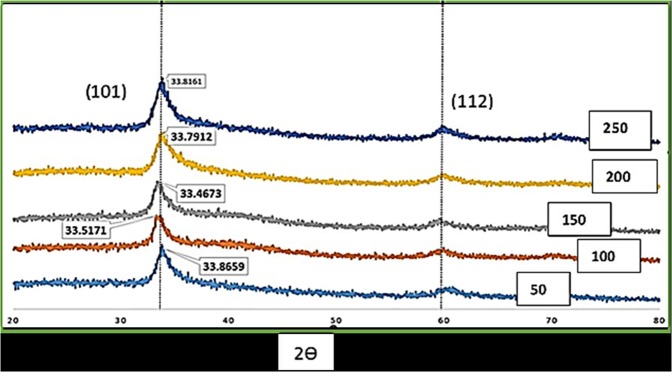
XRD patterns for various deposition powers.

Figure 8 shows the XRD patterns for as-sputtered WS_2_ films at different deposition powers where the 2ϴ angle ranges from 10° to 80°. All films exhibited two primary peaks of (101) and (112) orientations, indicating the polycrystalline nature of the as-sputtered WS_2_ films. The obtained XRD patterns of this study matched well with the standard XRD pattern documented in JCPDS-00-035-0651 file. All the films that are in 3R phase are semiconducting in nature^34^. The most intense peak was at 2ϴ = 33.8°, which corresponded to the preferred orientation of the (101) plane. The highest peak was found for two deposition powers, such as 50 and 150 W. However, the β value was almost the same for all variations. The β value represents the deterioration of crystalline properties of the film, and it is inversely proportional to the crystallite size (L)^35^.

The average particle size or crystallite size was calculated from the broadening of the (101) peak using the Scherrer equation^36^

1 $$L(hkl)=\frac{0.9\lambda }{{\rm{\beta }}\,\cos \,\theta }$$

where *L, λ, β*, and *θ* are the crystallite size, wavelength, FWHM, and angle between the incident and scattering planes, respectively.

**Figure 9:**
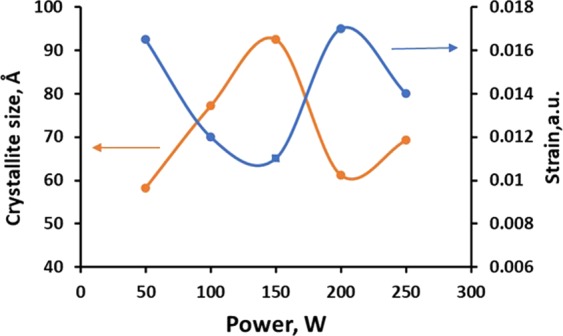
WS_2_ film crystallinity and strain with respect to sputtering powers.

Crystalline size was calculated and found to range from 93.97 Å to 62.43 Å. Grains with different relative orientations and positions create differences in phase variations when light waves are scattered by them. The large number of grains with different orientations result in the displacement of atom while forming crystal lattice. The lattice strains exhibited displacement of atoms from their original lattice positions, possibly due to the high energetic deposition. A low strain signifies high crystallinity^37,38^. Strain was calculated by the following relation:

2 $$\varepsilon =\frac{\beta }{4\,tan\theta }$$

where $\varepsilon$ is the strain. Figure 9 shows the variations in crystallinity and strain with respect to different deposition powers. At low deposition power, films exhibited high crystallite size, and the best results were observed at 150 W. As the power exceeded 150 W, the film crystallinity decreased, whereas strain increases. A deposition power of 200 W yielded the highest strain in the crystal structure. This phenomenon could be described as the impact of collisions between sputtered atoms. During sputtering, atoms with high energy bombard to the substrate. When the applied power is extremely high, atoms collide heavily during the formation of film on the substrate. This heavy collision between atoms deforms the crystallite structure at the edge, which increases strain to the film.

Strain and dislocation density of the film followed the same trend as reported by previous researchers. A high strain leads to the increased number of dislocation density in crystalline structure^35,38^. Thus, at 200 W deposition power, the dislocation density of as-grown WS_2_ thin film was the highest than that of the others. A high dislocation signifies that dislocations are separated by a distance greater than the interatomic distances. This unique property leads to a high optical bandgap of as-grown thin films^39^.

### Optical properties

**Figure 10:**
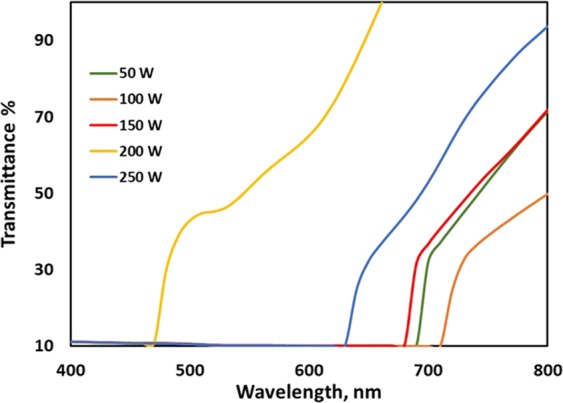
Optical properties of as-grown WS_2_ film: transmittance%.

The optical behavior of the as-deposited WS_2_ thin films at different RF powers was characterized by UV-VIS spectrometry. The absorbance of as-deposited films was calculated by the Beer–Lambert law, and the optical properties of the WS_2_ film are shown in Fig. 8. The Beer–Lambert law (or Beer’s law) is the linear relationship between the absorbance and concentration of an absorbing species. The general Beer–Lambert law is usually written as follows^40^:

3 $$A=a(\lambda )\ast b\ast c$$

where *A* is the measured absorbance, *a(λ)* is a wavelength-dependent absorption coefficient, *b* is the path length, and *c* is the analyte concentration. Transmittance is considered the inverse of absorbance^41^. The highest transmittance of as-grown WS_2_ film was observed for 200 W deposition power, which was over 60% at 550 nm. This result demonstrated the potential use of WS_2_ as a window layer material. Figure 10 depicts the transmittance of WS_2_ under different RF deposition powers.

A Tauc Plot is commonly used to determine the optical bandgap of semiconducting materials in the research community. A Tauc plot generally shows the quantity of the abscissa (the energy of the light spectrum) and the quantity (αhν)^1/n^ on the ordinate, where α is the absorption coefficient of the material^42,43^. The equation used to determine the bandgap is expressed as follows:

4 $$\alpha hv=A(hv-Eg{)}^{n}$$

Here, *α* is the measured absorption coefficient (cm^−1^) near the absorption edge, *A* is a constant, *hν* is the photon energy (eV), *Eg* is the optical bandgap (eV), and *n* is a constant. The value of n is determined from the nature of optical transition. n = 2 or 3 was adopted for indirect allowed and indirect forbidden transition, respectively. n = 1/2 or 3/2 was adopted for direct allowed and direct forbidden transition, respectively.

**Figure 11:**
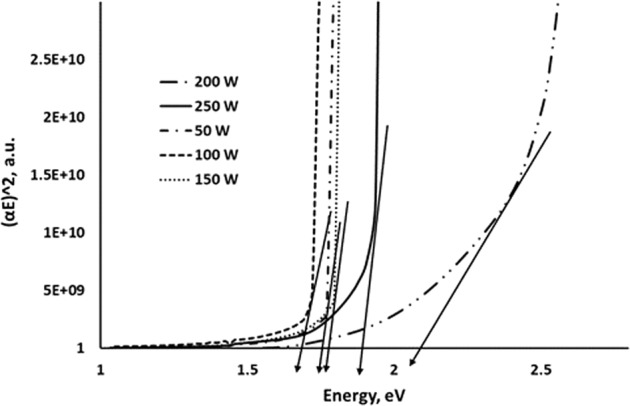
Tauc plot for as-deposited WS_2_ film for various deposition powers.

The optical bandgap of WS_2_ for various RF powers was evaluated by using the Tauc plot shown in Fig. 11. The optical bandgap of as-deposited WS_2_ thin films ranged from 1.7 eV to 2.2 eV. The results were well matched with the findings of a previous study^44^. These obtained results were highly desirable for photovoltaic materials and give strong recommendations to use WS_2_ as a photovoltaic material.

### Electrical properties

Hall effect measurement, invented by E.T Hall in 1879, is important for photovoltaic materials because it shows a semiconductor’s resistivity, mobility, and carrier density. Mobility is a measure of the epitaxial layer’s impurity content, quality, and homogeneity. Resistivity is the ratio of the electrical field to the current density^45^. Electrical properties of as-deposited WS_2_ films were subjected to Hall effect measurement. All the results, such as carrier concentration, hall mobility, resistivity, and conductivity. are summarized in Table 3.

**Table 3:** Hall effect measurement results of as-grown WS_2_.

RF Power (W)	Carrier concentration (cm^−3^)	Hall Mobility (cm^2^/V.s)	Resistivity (Ω-cm)	Conductivity type
50	8.89 × 10^18^	1.878	1.22	n
100	4.87 × 10^18^	2.38	1.41	n
150	9.85 × 10^18^	2.04	0.158	n
200	1.01 × 10^19^	1.26	0.135	n
250	1.45 × 10^19^	1.39	0.116	n

The carrier concentration and mobility of as-deposited films increased but resistivity decreased with the increase in RF power. The highest carrier concentration was observed at 250 W RF power with resistivity as low as 0.116 Ω-cm. At 200 W RF power, film resistivity was 0.135 Ω-cm with moderate mobility and high carrier concentration. During sputtering, a threshold energy for the release of an atom from the target was noted, below which the atom was not “sputtered.” When this threshold energy increased, it increased the deposition rate and carrier concentration. Therefore, conductivity is proportional to the product of mobility and carrier concentration. For instance, the same conductivity can be observed from a low carrier concentration but high mobility and vice versa^44,46^. When the deposition power increased, the carrier concentration increased without changing the conductivity of the film. Moreover, all the films exhibited n-type conductivity at all the applied deposition powers.

### Comparative study of WS_2_

Photovoltaic materials should be inexpensive and abundant. They should have good carrier concentration properties for both minority and majority carriers, low carrier recombination loss in grain boundaries, and adhesive to the surface^47^. Table 4 depicts the findings of a comparative study on the properties of existing photovoltaic materials (CdTe and CdS)^48^ and examined WS_2_. The photovoltaic optoelectrical properties of CdS and PVD-grown WS_2_ were similar. Moreover, from the perspective of material properties, WS_2_ is a non-toxic, abundant, adhesive, and semiconducting material. Thus, this study proposes to incorporate WS_2_ as a window layer in thin-film solar cells.

**Table 4:** Comparison of as-grown WS_2_ thin film with other photovoltaic materials.

Material	Band gap	Absorption coefficient	Carrier Concentration	Resistivity	Conductivity	Photovoltaic usage
CdTe	1.5	10^5^	10^14^	4.7	p-type	Absorber layer
WS_2_ (100 W)	1.75	10^5^	10^18^	1.96	n-type	—
CdS	2.45	10^4^	10^17^	0.1057	n-type	Window layer
WS_2_(200 W)	2.2	10^5^	10^17^	1.52	n-type	Proposed window layer

### Full device outcome

**Figure 12:**
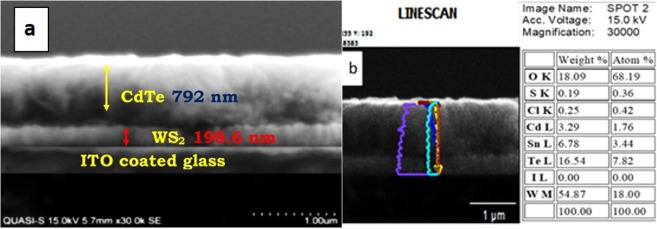
(**a**) SEM cross-sectional image of ITO/WS_2_/CdTe. (**b**) EDX line scan result of full cell.

**Figure 13:**
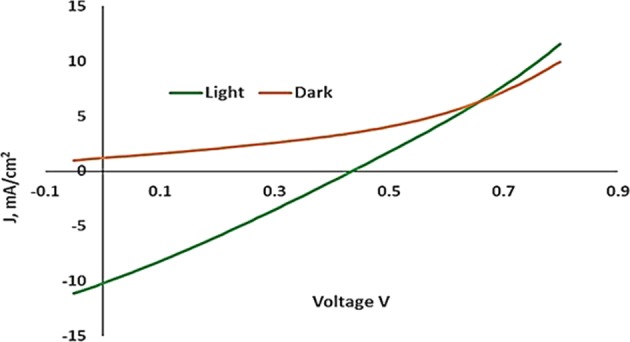
Dark and light IV curves of the fabricated solar cell.

To determine the reliability and evaluate performance, we incorporated WS_2_ thin film with 200 W deposition power as the window layer in CdTe/WS_2_ solar cell. Full ITO/WS_2_/CdTe/C/Ag devices were fabricated and examined. Figure 12(a) shows the SEM image of the as-deposited cell (three layers). EDX line scan (Fig. 12(b)) confirmed the atomic percentage of all elements present in the fabricated device. To evaluate the performance of the WS_2_-incorporated solar cell, it was characterized by an IV tester; it had an efficiency of 1.20%. Figure 13 shows the dark and light IV curves of the fabricated solar cell.

The device demonstrated an efficiency of 1.20% with J_sc_ = 10.45 mA/cm^2^, V_oc_ = 0.39 V, and FF = 29.42%. The dark IV curve showed that the leakage current was high in the fabricated cell, which indicated imperfections in the p-n junction. In the light IV curve, the fabricated cell presented increased series and parallel resistances under illumination, which resulted in lowered efficiency. All these drawbacks need to be addressed to achieve elevated efficiency.

## Conclusion

The first part of this study investigated the effect of RF deposition power on the sputter-grown WS_2_ film’s properties. Efforts were made to find the optimum film characteristics suitable for photovoltaic applications. In practice, pinhole reduction was achieved by optimizing the source-to-substrate distance. The adhesive test was also conducted to determine the best suited ones. For the range of growth conditions investigated here, deposited films were found to exhibit diffraction peaks corresponding to the (101) and (112) planes. All the films exhibited 3-R phase with granular morphology. By analyzing all the characteristics of as-deposited WS_2_ thin films grown at different RF powers, 200 W of RF deposition power was found suitable for using WS_2_ as a window layer material as it provided smooth surface morphology with an optical bandgap of 2.2 eV and high transmittance percentage to the visible light spectrum. At this deposition power, electrical properties such as carrier concentration and resistivity were 1.01 × 10^19^ cm^−3^ and 0.135 Ω-cm. respectively. These values agreed with the properties of the window layer material in solar cell devices.

The second part of the study was the successful demonstration of the incorporation of WS_2_ as photovoltaic material. With this optimized deposition parameter, WS_2_ thin film was successfully fabricated for the very first time as a window layer in CdTe solar cells. The new device (ITO/WS_2_/CdTe/Cu:C/Ag) exhibited V_oc_ = 0.39 V, J_sc_ = 10.45 mA/cm^2^, fill factor = 29.42%, and efficiency = 1.2%. The lowered efficiency was due to the optimized layer thickness of all layers and suitable ohmic contact, but these parameters were not investigated in this study. Nevertheless, this work successfully incorporated the low-cost non-toxic material WS_2_ in a solar cell device for the first time. Future work should focus on optimizing the device parameters, tuning the WS_2_ bandgap, analyzing defects in the interfaces, lowering cell resistance, and increasing the efficiency of solar cells.
